# Utilities of Patients with Hypertension in Northern Vietnam

**DOI:** 10.1371/journal.pone.0139560

**Published:** 2015-10-27

**Authors:** Thi-Phuong-Lan Nguyen, Paul F. M. Krabbe, Thi-Bach-Yen Nguyen, Catharina C. M. Schuiling-Veninga, E. Pamela Wright, Maarten J. Postma

**Affiliations:** 1 Department of Pharmacy, Unit of PharmacoEpidemiology&PharmacoEconomics (PE2), University of Groningen, Antonius Deusinglaan 1, 9713AV, Groningen, The Netherlands; 2 University of Groningen, University Medical Center Groningen, Department of Epidemiology, Groningen, The Netherlands; 3 Department of Health Economic, Ha Noi University of Medicine, Ha Noi, Vietnam; 4 Medical Committee Netherlands-Vietnam, Amsterdam, The Netherlands; 5 Institute for Science in Healthy Aging & healthcaRE (SHARE), University Medical Center Groningen (UMCG), Groningen, The Netherlands; National Cardiovascular Center Hospital, JAPAN

## Abstract

**Objectives:**

The study aims to inform potential cost-effectiveness analysis of hypertension management in Vietnam by providing utilities and predictors of utilities in patients with hypertension.

**Methods:**

Hypertensive patients up to 80 years old visiting the hospital were invited to participate in a survey using Quality Metric’s Short-form 36v2^TM^ translated into Vietnamese. Health-state utilities were estimated by applying a previously published algorithm.

**Results:**

The mean utility of the 691 patients interviewed was 0.73. Controlling for age, sex, blood pressure (BP) stage, and history of stroke, the utilities in older patients were lower than those in younger ones, and statistically significantly different between the extremes of youngest and oldest groups (p = 0.03). Utility in males was higher than in females (p = 0.002). As expected, patients with a history of stroke appeared to exhibit lower utilities than patients without such history, but the difference was not statistically significant (p = 0.73). Patients with more than three comorbidities did have lower utilities than patients without comorbidity (p = 0.01).

**Conclusions:**

Health-state utilities found among hypertensive patients in Vietnam were similar to those found in other international studies. It is suggested that lower of health-state utilities exist among those patients who were older, female or had more than three comorbidities in comparison with respective reference groups. However, further research for confirmation is required. The data from this study provide a potential reference on health-state utilities of hypertensive patients in Vietnam as an input for future cost-effectiveness analysis of interventions. Also, it may serve as a reference for other similar populations, especially in the context of similar environments in low income countries.

## Introduction

Hypertension and its complications represent key public health problems around the world especially among older adults. More than 40% of hypertensive person are older than 25 years [1]. Every year, 9.4 million deaths occur due to complications of hypertension [2]. Globally, diagnosis and treatment are relatively accessible and affordable, but even when patients are on treatment, there are health risks in the long term.

Different outcome measures can be applied to assess the effects of diagnosis and treatment, including health-state utilities, which are also core inputs for cost–effectiveness analysis of disease management. Notably, detailed measurements of utilities of hypertensive patients can support health economic evaluations of planned hypertension management programs. Currently, only limited information on health-state utilities of hypertensive patients is available for Asian countries, including Vietnam.

In many countries, the Short-form 36 version 2^TM^ (SF-36v2) questionnaire, as developed by Quality Metrics, has been applied to collect data on health-related quality of life (HRQoL) [3]. This instrument has also been applied for hypertensive patients in various countries [4–11], enhancing plausibility and acceptability of cross-country comparisons of HRQoL [9, 12, 13]. In Asia this instrument has already been validated in China, Singapore, Korea and the Philippines [14–17] which enhances plausibility that it is suitable for use in Vietnam.

In this study, we useSF-36v2^TM^ data from patients visiting a hospital out-patient clinic in an urban setting in Northern Vietnam to measure their HRQoL expressed in utilities. Furthermore, we aim to evaluate differences in health-state utilities related to characteristics of these patients to identify potential predictors.

## Methods

### Setting

We conducted the survey from April to May 2013 among patients attending the hypertension unit of the outpatient clinic in Thai Nguyen General Hospital; further details are described elsewhere [18]. The patients included new and existing cases coming to the outpatient clinic for the management of their hypertension.

### Participants

All confirmed hypertensive patients who were able to read and write, were no more than 80 years old and were willing to participate in this study were recruited. We invited patients to take the survey while they were waiting for examination; interviewers collected the data in the outpatient clinic. As one of our purposes was to investigate the relation between utilities and different patient characteristics, only records with full information were included in the analysis. Among 722 cases agreed to provide data, 712 records had full information on patients’ characteristic. Finally, 691 cases were complete and could be used to calculate health utility.

### Procedures and measurements

Five researchers of Thai Nguyen University of Medicine and Pharmacy were trained to interview patients. Notably, interviewers were trained in basic interview skills and structuring the interview in line with the contents of questionnaire. There was a practice section to make sure they have similar understanding and consistent applications of the questionnaire. Currently, only scare evidence and recommendation exist on what methods to use for measuring health utilities specially among hypertensive population. Notably, the algorithm produced by Brazier et al. used original valuation of SF-36v2^TM^ data, without potential errors related to mapping [19]. This model has also previously been shown to explain a reasonable share of variance (approximately 60%) and is considered sensitive in the measurement of impacts on health, especially in those cases when only relatively small differences in health are expected [19], as in the case of hypertension management. Given these advantages, this model was used to estimate health utilities based on SF-36v2^TM^ data in this study.

We applied the Quality Metric Short Form, SF-36v2TM, translated into Vietnamese, to collect data on health status. Health-state utilities were estimated by applying the previous derived algorithm [19]. For this model, eleven specific items of SF-36 were selected to estimate health-state utilities: in the physical domain, items number 3, 4 and 12 were included; in the role domain, items number 15 and 18; item number 32 in the social domain; items number 21 and 22 in the pain domain; items number 24 and 28 in the mental domain; and item number 27 in the vitality domain. Utilities were subsequently estimated based on responses elicited by standard gambling techniques [19]. Several steps to obtain health utilities values within the algorithm are presented in **S1 Appendix**.

During interviews we collected data on patient characteristics such as age, gender, occupation and ethnicity. Blood pressure (BP) was measured once at the time of the medical examination. Other health status parameters were collected from hard copies of medical records, including comorbidities such as diabetes, gout, heart valve disease, peripheral artery disease, chronic arthritis, lipid metabolism disorder, coronary artery disease, other chronic diseases, and history of stroke as a complication of hypertension.

### Statistical analysis

Descriptive listings, including percentages, frequencies, means and SDs for the various groups distinguished, were computed. We also applied independent t-tests, one-way ANOVA test to test the significance of differences in mean utilities between groups. Finally, multiple regression was applied to estimate influences of the individual variables on health-state utilities, using adjusted odds ratios (ORs).

### Ethical considerations

This study was approved by the ethical committee of the Thai Nguyen General Hospital. When patients were invited to join the study, the purpose was explained to them and they were told they could choose to join or not, and could stop the interview at any time. Those patients who did agree to provide the information gave verbal consent to the interviewer.

## Results

### Main characteristics of patients

The main characteristics of the 712 patients who met the inclusion criteria and joined the study are presented in Table 1. Patients’ ages ranged from 41 to 80 years; half belonged to the group 60–69 years and 56% was female. Only 10% of patients still worked for wages. Among the patients, 66% did not meet their target BP at the time of the survey, while 70% had comorbidities and 11% had a history of stroke. The components of comorbidities was presented in **S1 Table**.

**Table 1:** Characteristics of patients included in the study (n = 712).

Characteristics	Number (%)
**Age (years)**	
41–49	12 (1.7)
50–59	142 (19.9)
60–69	367 (51.5)
70–80	191 (26.8)
**Sex**	
Male	314 (44.1)
**Working status**	
Being retired or doing housework	637 (89.5)
Working and earning money	75 (10.5)
**Ethnicity**	
Kinh	651 (91.4)
Others	61 (8.6)
**Stage of BP at the time of survey**	
Target-BP	242 (34.0)
Stage one	360 (50.6)
Stage two	110 (15.4)
**Comorbidity**	
Comorbidity(ies)	500 (70.2)
**Stroke**	
History of stroke	78 (11)
**Health-state utility**	
Health-state utility (mean +/- SD (n = 691)	0.73 (+/- 0.14)

*Note*: Health utility was calculated in 691 patients, as syntax of transferring SF-36 data to utility did not work in 21 cases (3%) with missing value of any one of the items in the SF-36 form.

### Health-state utilities

Mean utility was estimated at 0.73 (+/- 0.14) in this hypertensive population. Mean health-state utilities were significantly different with respect to gender and age groups (all p<0.01). Table 2 shows that relatively higher BP appeared to be associated with lower health-state utility at 0.73, 0.73 and 0.71 in the groups with target BP, stage one and stage two, respectively. However, these differences were not statistically significant (p = 0.42). Regarding comorbidities or history of stroke, we did not find significant differences between the mean health-state utilities of these groups (Table 2).

**Table 2:** Distribution of health-state utilities by patients’ characteristic (n = 691).

Characteristics	Mean	Standard deviation	P value
**Age (years)**			0.004
41–49	0.787	0.16	
50–59	0.749	0.14	
60–69	0.730	0.14	
70–80	0.700	0.13	
**Sex**			0.005
Male	0.743	0.14	
Female	0.713	0.14	
**Stage of BP at the time of survey**			0.422
Target-BP	0.734	0.14	
Stage one	0.726	0.13	
Stage two	0.712	0.15	
**Comorbidity**			0.717
No comorbidity	0.729	0.14	
Comorbidity(ies)	0.725	0.14	
**History of stroke**			0.729
No stroke	0.727	0.14	
Stroke	0.721	0.15	

Controlling for other factors with adjusted ORs, we found that utilities in patients from 70 to 80 years old were lower than those of the younger groups; the difference between the oldest and youngest groups was statistically significant (Table 3; p = 0.03). Health-state utility in males was higher than in females even after adjustment for other factors (p = 0.002). Patients with a history of stroke exhibited an apparently lower health-state utility than patients without stroke, but the difference was not statistically significant (p = 0.79). Patients with more than three comorbidities had lower utilities than patients with no comorbidity, after adjustment (p = 0.01).

**Table 3:** Predictors of health-state utilities in hypertensive patients (n = 691).

	OR	95% CI		p
		Lower bound	Upper bound	
**Age (years)**				
41–49 (ref)				
50–59	-0.04	-0.12	0.04	0.34
60–69	-0.06	-0.13	0.02	0.16
70–80	-0.09	-0.17	-0.01	0.03
**Sex**				
Male (ref)				
Female	-0.03	-0.05	-0.01	0.002
**Stage of BP at the time of survey**				
Target-BP (ref)				
BP Stage one	-0.01	-0.03	0.01	0.43
BP stage two	-0.02	-0.05	0.02	0.32
**Stroke**				
No history of stroke (ref)				
History of stroke	-0.004	-0.04	0.03	0.79
**Comorbidity**				
No comorbidity (ref)				
One comorbidity	0.01	-0.02	0.03	0.53
Two comorbidities	-0.004	-0.03	0.03	0.81
Three comorbidities	-0.04	-0.08	0.02	0.20
> Three comorbidities	-0.11	-0.18	-0.03	0.01

## Discussion

This study aimed to fill gaps in the evidence on utilities for populations with high BP in low- and middle-income countries, in particular Vietnam. Accurate utility scores are crucial for a planned analysis of cost-effectiveness of disease management.

The mean utilities of our population of hypertensive patients are somewhat lower than the utilities of the general population in Vietnam. For example, the health-state utility of an older population in a rural community in Vietnam, measured by EQ-5D, was 0.88 while the utility in our population was 0.73 [20]. However, this comparison should be interpreted with caution as the studies used different instruments to measure utility [9, 12, 13]. Also, in comparison with other populations in Asian countries, estimated health-state utility is lower in our hypertensive population. For example, a general population in Singapore had mean health-state utility of 0.80 (+/-0.12), using the same method for health-state utility measurement [21].

Differences in utilities seem associated with age, gender, and a high number of comorbidities. However, no such statistically significant differences are found among BP-stages or with presence of history of stroke. Health-state utility among these hypertensive patients differed from an Australian study in which the utility of 0.63 was lower than the utility of 0.73 in our population, using the same instrument and method [22]. However, the population in that study consisted of prisoners and persons with an impaired mental health status, which may explain the lower health-state utilities. Results in other studies vary in health-state utility. For example, health-state utility in a hypertensive population (Beaver Dams study) was 0.72 or 0.83, measured with the Quality of Well-Being (QWB) and time trade-off (TTO) methods, respectively [13]. In a Swedish population with high BP, health-state utility was 0.73 when using a rating scale and 0.81 when using TTO [6]. In a Nigerian hypertensive population, it was relevantly lower at 0.35(+/-0.42), using the HUI3 method [23]. Again, these comparisons should be interpreted cautiously because different instruments and methods were applied [6]. Finally, in contrast with the aforementioned Nigerian study, in our study, health-state utilities do not significantly differ for different BP-levels. The methods of analysis between these two studies differed, with BP specified as a continuous variable in the Nigerian study but as a categorical one in our study [23]. In addition, in our study BP was measured only once at the time of examination; we do not know adherence to medication in this population or whether target-BP was achieved.

As expected, patients with a history of stroke have lower utilities than those without such a history in our study, however lacking statistical significance (likely due to relatively small numbers) [23]. Our finding that history of stroke does not significantly predicts utilities in our study population is in line with a similar study in a Swedish population [6]. Next to small numbers, a further reason may be that severity of health impacts and complications after stroke were relatively mild and all patients generally recovered.

In general, older patients have lower utilities than younger ones in our study. When controlling for sex and health status, the only significant difference found is between the oldest group and youngest group. Obviously and in general, utility decreases with age and in other studies it has indeed been quantified that each year of increasing age was reported to reduce health-state utility [22, 23]. In line with other studies, males have 0.03 higher mean health-state utility than females [22, 23]. We find an association between comorbidities and utilities, but this association is only manifest for patients having more than three comorbidities. In the Australian study, researchers reported that each increase in the number of comorbidities was associated with reduced utility [22]. It should be noted that our results appear without controlling for other variables that may affect HRQoL, such as body mass index, educational level, smoking, marital status, income/socioeconomic factors, serious events in the past, antihypertensive drugs, household position and awareness of hypertension [5, 9, 11, 20, 22, 24–27].

Regarding the limitations of the study, it should be mentioned that reliability and validity of applying the equation to obtain utilities from SF-36 in Vietnam should be investigated further, as it was developed for a Western population. We should be aware of potential similar problems as reported for other instruments [28, 29]. However, it has already been used in other Asian countries [21, 30] and currently does seem the optimal approach, most likely being acceptable for our measurements. In Vietnam, hypertension also occurs below the age of 40 years. However, we limited our study to 40 and over as younger patients were not available in the survey. Despite specific questions in the questionnaire, we were unable to get reliable estimates on the exact number of years since diagnosis of hypertension. Patients seemed unable to exactly recall year of diagnosis. Therefore, an analysis on the relationship between health utilities, duration, seriousness of progressive disease and burden of comorbidities overtime could not be performed in this study. Finally, due to scarce resources, capacity and funds, data collection lasted only two months, which may have influenced the type of patients included with respect to how they managed their hypertension in this specific hospital.

## Conclusions

Health-state utilities found among hypertensive patients in Vietnam were similar to those found in other international studies. It is suggested that lower of health-state utilities exist among those patients who were older, female or had more than three comorbidities in comparison with respective reference groups. However, further research for confirmation is required. The data from this study provide a potential reference on health-state utilities of hypertensive patients in Vietnam as an input for future cost-effectiveness analysis of interventions. Also, it may serve as a reference for other similar populations, especially in the context of similar environments in low income countries.

## Supporting Information

S1 Appendix Steps to quantify health utilities from SF-36 data. (DOCX)

S1 Dataset SF-36 data_Plos One paper. (SAV)

S1 Table Prevalence of comorbidities among 712 hypertensive patients. (DOCX)
