# Supplementary material for: Utilities of Patients with Hypertension in Northern Vietnam
Source: PLoS One. 2015 Oct 27;10(10):e0139560. doi: 10.1371/journal.pone.0139560 (PMC4623979; doi:10.1371/journal.pone.0139560)
Supplement: S1 Appendix — (DOCX) [file pone.0139560.s001.docx]

**Appendix S1. Steps to quantify health utilities from SF-36 data^[[1]](#endnote-1)^**

First, values of SF-6D were derived from SF-36 by recoding

| **Values of SF-36** | **Values of SF-6D** |
| --- | --- |
| ***For physical functioning dimension*** |  |
| (sf3=3) AND (sf4=3) AND (sf12=3) | SFPhys = 1 |
| (sf3=1) OR (sf3=2)) AND (sf4=3) AND (sf12=3) | SFPhys = 2 |
| (sf4=2) AND (sf12=3) | SFPhys = 3 |
| (sf4=1) AND (sf12=3) | SFPhys = 4 |
| (sf12=2) | SFPhys = 5 |
| (sf12=1) | SFPhys = 6 |
| ***For role limitations dimension*** |  |
| (sf15=5) AND (sf18=5) | SFRole = 1 |
| ((sf15=1) OR (SF15=2) OR (Sf15=3) OR (sf15=4)) AND (sf18=5) | SFRole = 2 |
| ((sf18=1) OR (SF18=2) OR (Sf18=3) OR (sf18=4)) AND (sf15=5) | SFRole = 3 |
| ((sf15=1) OR (sf15=2) OR (sf15=3) OR (sf15=4)) AND ((sf18=1) OR (sf18=2) OR (sf18=3) OR (sf18=4)) | SFRole = 4 |
| ***For social functioning dimension*** |  |
| (sf32=5) | SFSocial = 1 |
| (sf32=4) | SFSocial = 2 |
| (sf32=3) | SFSocial = 3 |
| (sf32=2) | SFSocial = 4 |
| (sf32=1) | SFSocial = 5 |
| ***For bodily pain dimension*** |  |
| (sf21=1) AND (sf22=1) | SFPain = 1 |
| ((sf21=2) OR (sf21=3) OR (sf21=4) OR (sf21=5) OR (sf21=6)) AND (sf22=1) | SFPain = 2 |
| (sf22=2) | SFPain = 3 |
| (sf22=3) | SFPain = 4 |
| (sf22=4) | SFPain = 5 |
| (sf22=5) | SFPain = 6 |
| ***For mental health dimension*** |  |
| (sf24=5) AND (sf28=5) | SFMental = 1 |
| (sf24=4) AND (sf28=5) | SFMental = 2 |
| (sf24=4) AND (sf28=4) | SFMental = 2 |
| (sf24=5) AND (sf28=4) | SFMental = 2 |
| (sf24=3) AND ((sf28=3) OR (sf28=4) OR (sf28=5)) | SFMental = 3 |
| (sf28=3) AND ((sf24=4) OR (sf24=5)) | SFMental = 3 |
| (sf24=2) AND ((sf28=2) OR (sf28=3) OR (sf28=4) OR (sf28=5)) | SFMental = 4 |
| (sf28=2) AND ((sf24=3) OR (sf24=4) OR (sf24=5)) | SFMental = 4 |
| (sf24=1) | SFMental = 5 |
| (sf28=1) | SFMental = 5 |
| ***For Vitality dimension*** |  |
| (sf27=1) | SFVital = 1 |
| (sf27=2) | SFVital = 2 |
| (sf27=3) | SFVital = 3 |
| (sf27=4) | SFVital = 4 |
| (sf27=5) | SFVital = 5 |
| ***Interaction term (MOST)*** |  |
| (SFPhys=4) OR (SFPhys=5) OR (SFPhys=6) OR (SFRole=3) OR (SFRole=4) OR (SFSocial=4) OR (SFSocial=5) OR (SFPain=6) OR (SFMental=4) OR (SFMental=5) OR (SFVital=4) OR (SFVital=5) | Most=1 |

Second, distributing values for SF-6D to estimate health utility score

| Values of SF-6D | Values for the algorithms |
| --- | --- |
| ***For physical functioning dimension*** |  |
| (SFPhys=1) | pf=0 |
| (SFPhys=2) OR (SFPhys=3) | pf = -.035 |
| (SFPhys=4 ) | pf = -.044 |
| (SFPhys=5) | pf = -.056 |
| (SFPhys=6) | pf=-.117 |
| ***For role limitations dimension*** |  |
| (SFRole=1) | rl=0 |
| (SFRole=2) OR (SFRole=3) OR (SFRole=4 ) | rl = -.053 |
| ***For social functioning dimension*** |  |
| (SFSocial=1) | sc=0 |
| (SFSocial=2) | sc=-.057 |
| (SFSocial=3) | sc=-.059 |
| (SFSocial=4) | sc=-.072 |
| (SFSocial=5) | sc=-.087 |
| ***For bodily pain dimension*** |  |
| (SFPain=1) | pn=0 |
| (SFPain=2) OR (SFPain=3) | pn = -.042 |
| (SFPain=4 ) | pn = -.065 |
| (SFPain=5) | pn = -.102 |
| (SFPain=6) | pn=-.171 |
| ***For mental health dimension*** |  |
| (SFMental=1) | mh=0 |
| (SFMental=2) OR (SFMental=3) | mh = -.042 |
| (SFMental=4 ) | mh = -.100 |
| (SFMental=5) | mh = -.118 |
| ***For Vitality dimension*** |  |
| (SFVital=1) | v =0 |
| (SFVital=2) OR (SFVital=3) OR (SFVital=4 ) | v = -.071 |
| (SFVital=5) | v = -.092 |
| ***Interaction term (MOST)*** |  |
| (MOST=0) | mst=0 |
| (MOST=1) | mst=-.061 |

Notes: sf from 1 to 36 was in accordance with order of question in the SF-36 questionnaire, for example sf12 is question number 12 in SF-36 form.

Finally, calculating health utilities of patients was done using below formula:

Health utility = 1 + pf+rl+sc+pn+mh+v+mst

1. The algorithm was derived from Brazier J, Roberts J, Deverill M The estimation of a preference-based measure of health from the SF-36. J Health Econ. 2002; 21: 271-292. [↑](#endnote-ref-1)
