# Supplementary material for: Utilities of Patients with Hypertension in Northern Vietnam
Source: PLoS One. 2015 Oct 27;10(10):e0139560. doi: 10.1371/journal.pone.0139560 (PMC4623979; doi:10.1371/journal.pone.0139560)
Supplement: S1 Table — (DOCX) [file pone.0139560.s003.docx]

**Table S1. Prevalence of comorbidities among 712 hypertensive patients**

| **Comorbidities** | **Number of patients** | **Prevalence (%)** |
| --- | --- | --- |
| Diabetes | 9 | 1.3 |
| Gout | 18 | 2.5 |
| Heart valve disease | 34 | 4.8 |
| Peripheral artery | 4 | 0.6 |
| Chronic arthritis | 157 | 22.1 |
| Lipid metabolism disorder | 188 | 26.4 |
| Disease related to nervous system | 61 | 8.6 |
| Coronary artery | 35 | 4.9 |
| Endocrine | 4 | 0.6 |
| Other internal medical diseases | 245 | 34.4 |
